# Role of Regulatory T Cells in Skeletal Muscle Regeneration: A Systematic Review

**DOI:** 10.3390/biom12060817

**Published:** 2022-06-11

**Authors:** Jaciara F. G. Gama, Rayza D. Romualdo, Mayara L. de Assis, Luana M. de Oliveira, Thereza Quírico-Santos, Luiz A. Alves, Jussara Lagrota-Candido

**Affiliations:** 1 Laboratório de Imunopatologia, Departamento de Imunobiologia, Universidade Federal Fluminense, Niterói, Rio de Janeiro 24210-201, Brazil; bmd_gomes@yahoo.com.br (J.F.G.G.); rayzadr@id.uff.br (R.D.R.); assismayara@id.uff.br (M.L.d.A.); luana_oliveira@id.uff.br (L.M.d.O.); tquirico@id.uff.br (T.Q.-S.); 2 Laboratório de Comunicação Celular, Fundação Oswaldo Cruz (FIOCRUZ), Instituto Oswaldo Cruz (IOC), Rio de Janeiro 21040-900, Brazil; alveslaa@gmail.com

**Keywords:** T regulatory cell, skeletal muscle, muscle repair, regeneration, regulatory microenvironment

## Abstract

Muscle injuries are frequent in individuals with genetic myopathies and in athletes. Skeletal muscle regeneration depends on the activation and differentiation of satellite cells present in the basal lamina of muscle fibers. The skeletal muscle environment is critical for repair, metabolic and homeostatic function. Regulatory T cells (Treg) residing within skeletal muscle comprise a distinct and special cell population that modifies the inflammatory environment by secreting cytokines and amphiregulin, an epidermal growth factor receptor (EGFR) ligand that acts directly upon satellite cells, promoting tissue regeneration. This systematic review summarizes the current knowledge regarding the role of Treg in muscle repair and discusses their therapeutic potential in skeletal muscle injuries. A bibliographic search was carried out using the terms Treg and muscle regeneration and repair, covering all articles up to April 2021 indexed in the PubMed and EMBASE databases. The search included only published original research in human and experimental animal models, with further data analysis based on the PICO methodology, following PRISMA definitions and Cochrane guidelines.

## 1. Introduction

Skeletal muscle constitutes 40% of the total body composition, essential for postural support, movement, breathing, and temperature body control, also playing important structural and biochemical functions in daily living [1]. The muscle tissue displays a high regeneration capacity due to the activation of myogenic precursors known as satellite cells, a population of postnatal muscle stem cells (MuSCs) located between the sarcolemma and basal lamina of muscle fiber [2]. Following muscle injury, mitotically quiescent satellite cells are readily activated and proliferate to either replenish the satellite cell niche or differentiate into myoblasts [3]. Satellite cell differentiation is mainly regulated by the expression of myogenic regulatory factors (MRFs), including the myogenic factor 5 (Myf5), myogenic differentiation (MyoD), myogenic regulatory factor 4 (Mrf4), and myogenin, all considered critical for the terminal differentiation of MuSCs into myotubes [4,5]. Crucially, efficient muscle repair depends on the interaction between myogenic stem cells and an adequate muscle microenvironment capable of providing cellular signaling for efficient tissue regeneration and remodeling [6].

Skeletal muscle presents essential tissue plasticity, capable of efficient regeneration after daily injuries caused by several contraction and relaxation cycles [7]. Some processes lead to extensive or repetitive insult due to genetic defects, such as muscular dystrophies, in which chronic myofiber loss and satellite cell pool exhaustion dampen efficient tissue repair, with further muscle tissue replacement by connective tissue and fibrosis [8,9]. Inflammatory events in the tissue microenvironment following muscle injury are critical for adequate muscle repair and/or persistent fibrosis [10]. The muscle injuries themselves induce the release of biologically active molecules into the extracellular space, attracting inflammatory cells, which actively modulate the activation, proliferation, and differentiation of MuSCs [11]. Muscle tissue repair appears to take place in consecutive but interconnected waves comprising degeneration, inflammation, regeneration, maturation-remodeling, and functional recovery. Since the previous wave influences the next event, it is important to understand all distinct cellular and molecular aspects involved in each event to allow the development of therapies capable of improving tissue repair [12].

In the initial acute injury phase, a pro-inflammatory response is mediated by the transient recruitment of neutrophils and M1 macrophages from the innate immune system; however, a change to the M2 macrophage profile with an anti-inflammatory action is of paramount importance [13,14]. Muscle repair efficiency depends on factors initiated by the innate immune response characterized by a transient wave of neutrophils followed by a more persistent infiltration of macrophages, a condition for effective muscle regeneration [15]. M1 and M2 macrophages are temporally regulated during muscle injury repair and can promote either further injury or repair, according to macrophage phenotype. During the initial injury phase, M1 macrophages recruited from a pool of circulating monocytes infiltrate the lesion for clearance of debris and apoptotic or necrotic cells and are activated upon tumor necrosis factor (TNF-α) stimulation [13]. M1 macrophages promote pro-inflammatory responses through the release of cytokines and can lyse muscle cells and damage tissue through inducible nitric oxide synthase (iNOs) secretion [16,17]. Subsequently, an increase in M2a or M2c macrophages producing anti-inflammatory cytokines and arginase is responsible for decreasing the M1 macrophage response and influencing the course of tissue injury by promoting muscle growth and regeneration [17]. The replacement of M1 by M2 macrophages induces a change from a pro-inflammatory to an anti-inflammatory profile, required to promote correct tissue repair and regeneration [13]. In this context, the muscle microenvironment exhibits an important physiological role with the production of cytokines and proteases capable of providing the remodeling milieu of the muscle tissue. Another important feature in the muscle microenvironment is the production of IL-10, which protects the organism from autoimmunity [18,19].

In the last two decades, T lymphocytes have also been described as playing an important role in skeletal muscle repair. T cells, depending upon the microenvironment and how they are stimulated, can produce distinct patterns of cytokines that differently modulate the activity of other cells present within muscle tissue, such as macrophages, satellite cells, and fibroblasts [20]. In vitro studies show that T-cell supernatant possesses both mitogenic and chemoattractant functions on myogenic precursors [21,22]. CD8^+^ lymphocytes facilitate macrophage chemokine protein-1 (MCP1) secretion, thereby increasing migration of Gr1^high^ macrophage subset and inducing myoblast proliferation in a cardiotoxin-induced sterile muscle injury model [23]. Interestingly, CD4^+^ and CD8^+^ lymphocytes have been associated with muscle regeneration impairment in humans with polymyositis and dermatomyositis, mainly by increasing the muscle damage through the IL-15 induction by inflammatory cytokines (e.g., IFN-γ, IL-1β, and TNF-α) [24,25]. CD4^+^ and CD8^+^ T cells have also been associated with muscle degeneration and fibrosis in the mdx mice, a natural mutant that does not express dystrophin and Duchenne muscular dystrophy (DMD) homolog, by induction of specific antigen activation of CD44^+^ cells homing into the damaged tissue [26,27,28]. Myotoxic events have been associated with the oligoclonal expansion of TCR-αβ T lymphocytes and the induction of diaphragm and cardiac fibrosis [26,29]. However, only some years later, regulatory T cells (Treg) were observed in dystrophic muscle tissue [30,31,32] and were considered as promoters of repair and tissue regeneration [33].

Treg cells express Foxp3 protein as their main cell marker. Muscle Treg cells participate in the change from a pro-inflammatory to an anti-inflammatory subset and act by stimulating muscle satellite cells to proliferate and migrate mainly through amphiregulin release [33,34]. The current systematic review was carried out to summarize Treg cell contributions in different muscle injury models and address potential therapeutic applications.

## 2. Materials and Methods

### 2.1. Review Questions

Our main aims were to (1) assess the role of regulatory T cells in different reported muscle injury models, (2) evaluate putative in vitro actions of regulatory T cells on myoblasts, and (3) discuss the effects of regulatory T-cell modulation in distinct muscle lesion animal models.

### 2.2. Search Strategy

An extensive systematic search carried out on the database PubMed and Embase (up to 23 April 2021) was conducted by guidelines of the “PRISMA Declaration for reporting systematic reviews and meta-analysis”. For searching Pubmed and Embase databases, the MeSH and Emtree strategies were used, including synonyms and plural terms with the most relevant keywords: “T-Lymphocytes, Regulatory”, “Foxp3”, “Skeletal muscle”, “Muscle injury”, “Regeneration”, and “Repair”. For search in Pubmed database were selected “all fields” and PICO strategy for Embase database. Details of the selected search terms and search procedures that were used in the databases on an individual basis are available in Table 1.

### 2.3. Selection

The Mendeley platform was used to access the PubMed and Embase databases, and duplicates were checked using Mendeley Desktop. Studies that were not original papers, such as reviews, editorials, comments, and letters, were excluded. Articles are written in another language than English or Portuguese were also excluded. Primary screening of titles and abstracts was performed to exclude articles with cardiac or smooth muscle and muscle injuries resulting from infectious and/or autoimmune diseases. Two different investigators (R.D.R. and M.L.A.) performed the search and evaluated the articles independently. Eventual disagreements were discussed with a third investigator (J.F.G.G.) to reach a final consensus. In addition, three first literature reviews of Google Scholar from top hits referring to skeletal muscle regeneration/repair and regulatory T cells were also reviewed for further inclusion of potentially relevant studies not found by the initial search.

### 2.4. Bias Analysis

Assessment of methodological quality of the studies was performed by four independent reviewers (J.F.G.G., R.D.R., M.L.A., and L.M.O.) according to the criteria described on the SYRCLE’s Risk of Bias tool (Systematic Review Centre for Laboratory animal Experimentation) described previously [35]. Cases of disagreement in rating were discussed with the senior investigator (J.L.C.) to achieve a consensus for a final score. The domains (D) described previously by Hoojmans and coworkers (2014) were used to assess the risk of bias: selection, performance, attrition, detection, reporting, and other biases. The response was defined as “Low risk”, “High risk”, or “Unclear risk” of bias for each article analyzed. Studies that cited the use of randomization to generate the allocation sequence (D1) but did not detail the methodology were considered an uncertain risk of bias, and studies that did not mention randomization were classified as high risk of bias. Baseline characteristics (D2) were scored as low risk of bias when it was defined gender and age of groups as well as detailed information about the method of injury. Regarding the allocation concealment bias (D3), the study was considered with low risk when using appropriate control groups. Concerning performance bias, the absence of description on random housing (D4), and blinding of caregivers and/or researchers (D5) concerning the intervention of each animal during the experimental procedure were classified as unclear risks. Original articles that did not provide enough information whether the whole experimental group was used or how investigators randomly used an animal for outcome assessment (D6) were scored as high risk. Original articles that reported a blind evaluator (D7) for analysis of histology and immunohistochemical outcomes were scored with a low risk of bias. Unclear risk of completeness of outcome (D8) was scored when authors did not report the numbers in each intervention group used at the start and end of the experiment and also reasons for attrition or exclusions. Selective outcome reporting domain (D9) was scored as low when the results of all outcome variables assessed were reported and discussed completely. Another risk of bias (D10) is scored green when they presented detailed methodology statistics, animal ethics committee approval, and conclusions were supported by the experimental results.

## 3. Results

### 3.1. Search Results

**Figure 1:**
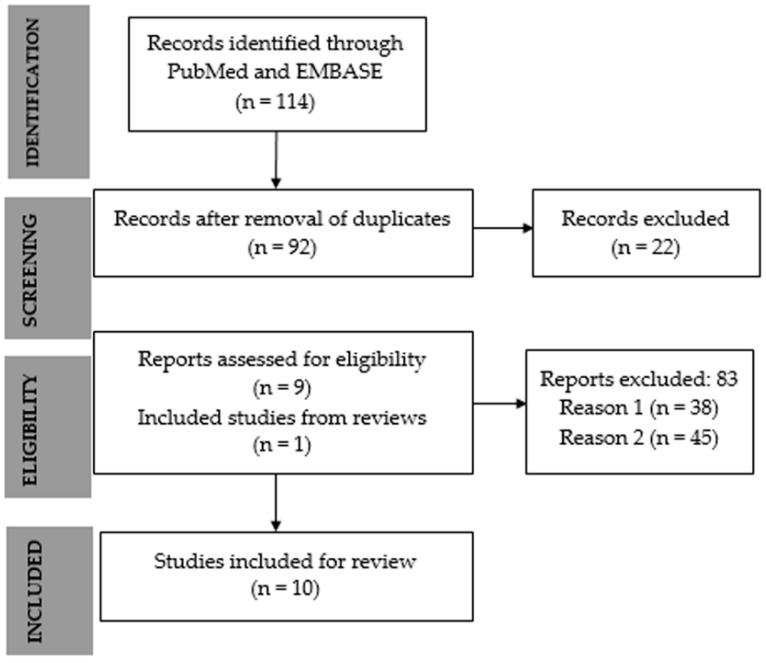
Flowchart of search strategy and selection. Reason 1: type of article; Reason 2: content out of revision topic.

There were 114 articles identified by searching the PubMed and Embase databases: 81 from Pubmed and 33 from Embase. In total, 92 articles remained for analysis after checking and exclusion of 22 duplicates. After a detailed analysis of eligibility, 83 articles were further excluded for the following reasons: (1) type of the article, 38 articles were a review, editorial, comments, letters, or chapters and written in a language other than English or Portuguese; (2) content of the article, 45 articles were excluded for not being related to the theme of this review as articles about cardiac or smooth muscle, muscle injuries resulting from infectious and autoimmune diseases. After reviewing references from the first three literature reviews found in Google Scholar’s relevance top, one article was further included. Additionally, 10 articles that met all inclusion criteria were identified and included in the present systematic review. The flowchart of the selection process for the selected studies is shown in Figure 1.

### 3.2. Quality Assessment

**Figure 2:**
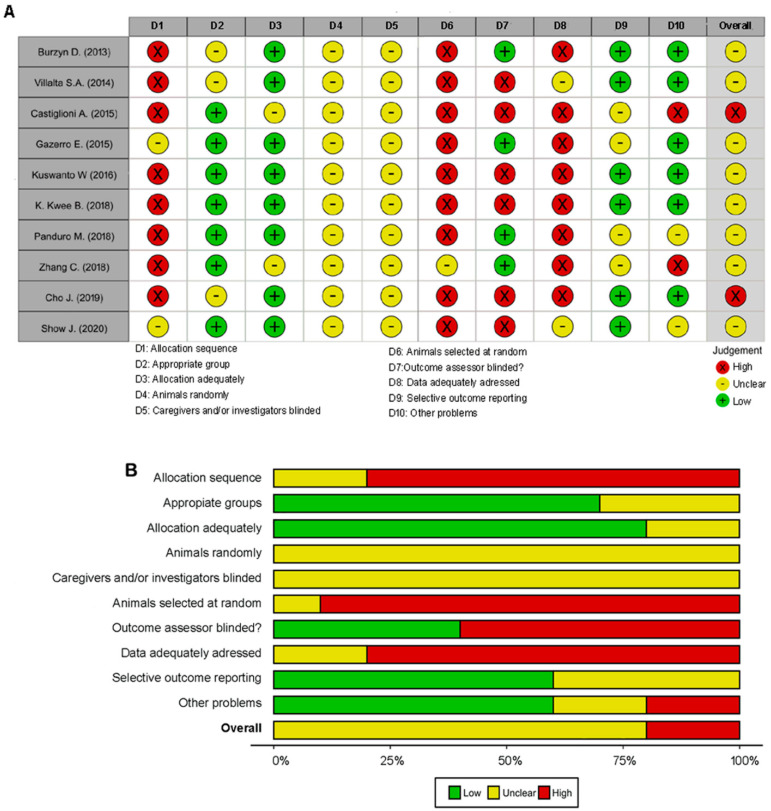
Risk of bias assessment. The Systematic Review Centre for Laboratory Animal Experimentation was used to assess the risk of bias in the included studies. Green—low risk of bias; red—high risk of bias; yellow—unclear risk of bias. (**A**) Individual studies of bias analysis based on SYRCLE methodology. (**B**) An overview of results.

The methodological rigor was assessed through bias analysis as described in Methods. During the critical reading of the articles, we evidenced that some articles did not present, according to Hoffmans’ guidelines, enough information for an adequate analysis of the bias that could affect the classification [35]. None of the articles showed detailed performance bias and were therefore classified as unclear risk. Individual studies based on SYRCLE bias analysis are detailed in Figure 2A and the overview of results in Figure 2B.

### 3.3. Study Characteristics

The initial publication of selected articles on the subject of this review dates from 2013 with at least one annual publication until 2021, with the exception of publications in 2017 and 2020. We verified that six studies were conducted in the United States, with four being from the same research group [33,36,37,38,39]; two studies in Italy [40,41]; and two in China [42,43]. All studies used mice as animal model to analyze Treg levels in skeletal muscle lesions from dystrophic mdx mice (three studies), cryo-injury (two studies), or lesion induced by cardiotoxin (five studies), ischemia (one study), and contusion (one study). One study [33] used more than one type of injury induction model.

### 3.4. Treg Is Increased in Injured Muscle

Seven out of ten articles analyzed Treg levels in the injured muscle (Table 2). Treg CD4^+^ Foxp3^+^ cells were found to have an increase in both acute injuries induced by cryo-injury and cardiotoxin, and chronic injuries in mdx dystrophic mice. Treg cells have been accumulated in skeletal muscle shortly after the injury, with in situ clonal expansion and maintenance of high levels throughout the repair process. It was consistently observed that muscle Treg cells secreted IL-10 cytokine in the following injury models: cryo-injury, cardiotoxin-induced injuries, and mdx mice [33,36,38,39,40,42,43]. Growth factor Amphiregulin (AREG) released by muscle Tregs could have acted directly on MuSCs in vitro and has improved muscle repair in vivo, as evidenced in cryo-injury and cardiotoxin models [33,36,38,40,42,43]. Furthermore, the transcriptome of muscle Treg has been shown to be distinct from that of lymphoid-organ Treg and is established in the skeletal muscle microenvironment, which suggests a unique tissue-specific profile [33]. Just one study performed Treg analysis on muscle damage in humans DMD/BMD biopsies [39] reported that high Treg frequency was associated with an IL-10 secretion increase.

### 3.5. Do Treg Cells Modify Muscle Repair?

Among 10 studies, seven used intervention tools to increase or decrease Treg cells to analyze their influence on muscle repair. A summary of interventions and their respective characteristics is presented in Table 3.

Expansion of Treg cells was obtained by inoculation of IL-2/IL2 receptor complex [33,39] or IL-33 [38] in mdx dystrophic mice. Blockade of the extracellular ATP/P2X purinergic signaling pathway by administration of periodate-oxidized ATP (oATP) also induced accumulation of Treg cells in the skeletal muscle [41]. These studies showed that the high frequency of Tregs induced efficient muscle repair independently on the injury model used. Accumulation of Treg cells induced by administration of oATP and IL-2/IL2 receptor complex mitigated muscle inflammation. IL-33 administration in old mice decreased the deficit of Treg cells in cryo-lesion-induced muscle injury and improved muscle regeneration; however, they did not analyze inflammatory parameters [38]. In addition, mdx mice were crossed with transgenic mice carrying rearranged TCR genes from a muscle Treg clone and showed an accumulation of these cells in dystrophic muscle [36]. Although leukocyte infiltration was not significantly different between wild-type and transgenic mdx, muscle regeneration was upregulated in transgenic animals.

A decrease in Treg levels was associated with worsening muscle damage and impaired regeneration, maintenance of the inflammatory profile for longer days, and fibrosis more intense. Ablation of Treg cells in dystrophic mice was achieved by anti-CD25 treatment in 2–3-week-old mdx mice before the peak of inflammation [33,39]. In addition, to specifically deplete Tregs, the authors used DEREG (DEpletion of REGulatory T cells) mice, which carry a diphtheria toxin receptor (DTR) under the control of Foxp3 regulatory elements [45,46] and applications of diphtheria toxin starting at the day of induction of cryo-injury or cardiotoxin-induced lesion [33,39,44]. Experiments in DEREG mice showed that the absence of Treg induced an increase in interferon-gamma (IFN-γ) production by conventional T and NK cells and enhanced M1 macrophages activation [39,44]. In addition, a mouse lacking ST2 (IL-33 receptor) specifically on T lymphocytes was used to decrease the migration of Treg cells into the injured muscle. The induction of cryo-lesion in such animals confirmed the importance of Treg cells for controlling inflammation and inducing muscle regeneration [38]. Schou and coworkers (2021) used knockout PD-1 mice with reduced numbers of Treg cells. PD-1 belongs to the CD8 family and has a role in Treg induction in the periphery [42]. Contused muscles of these animals showed greater persistence of inflammation and delayed muscle regeneration, thus confirming the importance of Treg in another model of muscle injury. However, Treg’s conditioned medium, inoculated together with injectable alginate biomaterial for slow release, did not induce substantial effects on ischemic muscle in vivo [37].

Among ten articles, only four presented in vitro assays to demonstrate Treg functions related to skeletal muscle repair (Table 4). MuSCs isolated of cardiotoxin-injured muscle from Treg-depleted mice (DT-treated DTR) presented a decreased colony-forming capacity evaluated in clonal myogenesis assays [33]. Castiglioni et al. (2015) co-cultivated MuSCs with iTreg obtained from the incubation of lymph node CD4^+^ T cells with TGF-β and IL-2. The authors observed that the number of MuSCs was significantly higher in the presence of iTreg despite a mild increase in BrdU^+^ percentage of cells. Longer interaction with induced Treg (iTregs) delayed their terminal myogenic differentiation, evidenced by the fusion index [40]. Kwee et al. (2018) showed in vitro the influence of factors secreted by T lymphocyte subtypes on angiogenesis and myogenesis [37]. The authors stimulated CD4^+^ T lymphocytes from mouse lymph nodes and spleen in the presence of different cytokines. Conventional Treg cells were induced by incubation with IFN-γ, TGF-β, and IL-2. However, secreted factors from Treg cells showed moderate or no effect on angiogenesis and myogenesis in vitro [37]. To investigate how Treg are attracted to muscle, Zhang et al. (2018) detected chemokines and chemokine receptors within the injured muscle. The authors found that CCL3 was overexpressed in the cryo-injured muscle and exerted significant chemotactic stimuli for conventional Treg migration on transwell plates and that anti-CCR1 antibody reversed such migration [43].

## 4. Discussion

A pioneering study from Diane Mathis’s group [33] showed that Treg cells could directly or indirectly influence the repair of aseptic muscle injuries. However, previous works that did not fulfill the research requirements of this systematic review have already shown that Treg cells could participate in muscle tissue injury [47,48,49,50]. Indeed, our group highlighted the accumulation of CD25^+^ T lymphocytes in the draining lymph nodes of mdx muscle mice at 12 and 24 weeks, corresponding to the period when inflammation is under control. Such a result suggested that activation of CD25^+^ T lymphocytes was associated with the control of the inflammatory response in the murine model of DMD [31]. CD25 is a marker of regulatory T lymphocytes, although functional experiments are essential to confirm the regulatory function of such cells. Later, the demonstration that Foxp3 was an exclusive marker for Treg [51,52] made it possible to ascertain the presence of Treg cells in the mdx muscle [30,32]. Interestingly, DMD patients with intense inflammation and progressive fibrosis were found to have high levels of mRNA for IL-17 but low Foxp3 expression [53].

The process of muscle regeneration can vary considerably among different types of injury, such as mechanical, physical, and chemical injuries for the study of acute muscle injuries. Induction of ischemia and contusion are often used as physical injuries. Cardiotoxin, a myotoxin extracted from snake venoms, was the most commonly used model due to extensive myolysis of myofibers when injected intramuscularly while preserving satellite cells [54,55]. However, to quantify histological alterations of muscle injury, some authors preferred to use the cryo-lesion model mainly because the area of injury is clearly delimited and homogeneous [33,38]. Cryo-injury destroys muscle fibers and also satellite cells, although the injury may attract MuSCs to repair [56].

The mdx4cv mouse produced from mdx shows alterations in other dystrophin isoforms [57]. The mdx model is widely used to study muscle regeneration because it presents a more benign disease with mild clinical signs, a period of intense myonecrosis between 3 and 8 weeks followed by cycles of regeneration, but after 12 weeks, although being capable of controlling inflammation, there is evident increase deposition of connective tissue [31,57,58].

Normal muscle presents low numbers of muscle Treg cells [33], but in all models of acute and chronic muscular injuries (Table 1), there is a consistent expansion of muscle Treg cells. Evidence of oligoclonal expansion of muscle Treg cells suggests that DAMP recognition is associated with muscle damage, although still a matter of debate. In this regard, Treg cells expressing TCR receptor from muscle Treg clone have a preferential migration into injured muscle, showing TCR-dependent management [36]. It is important to highlight that aging has impaired muscle tissue repair and decreased the ability of Tregs to accumulate and settle in the muscle niche [38].

**Figure 3:**
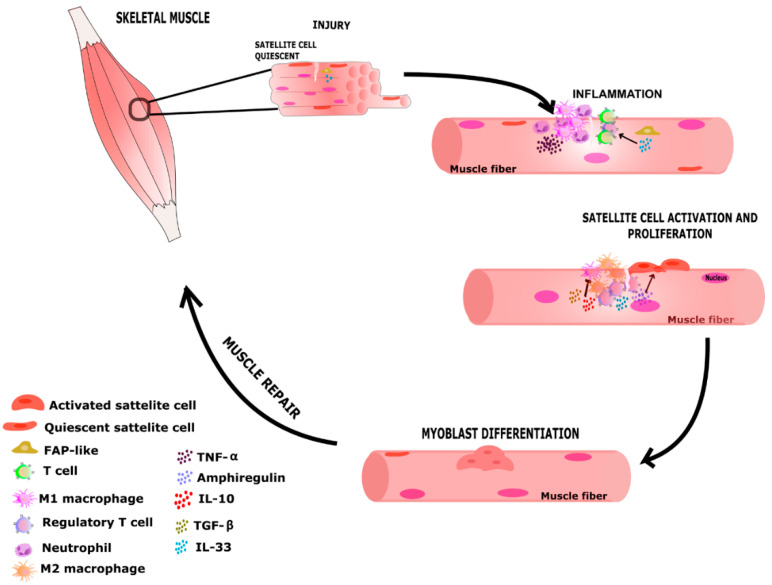
Illustrative image of events related to skeletal muscle acute injury. Following an acute muscle injury, a series of events are triggered in a coordinated manner to restore tissue function and promote muscle regeneration. The muscle inflammatory environment plays an important role in influencing tissue repair with participation of Treg, FAP-like, satellite cells, and soluble factors such as cytokines (e.g., IL-10, TGF-β, IL-33, TNF-α) and growth factors (e.g., AREG). Disturbance in those events could lead to an imbalance in the regeneration process and inefficient repair of muscle tissue. FAP-like—Fibro-adipogenic precursor-like.

The importance of muscle Tregs is evident since their upregulation induces muscle regeneration and their ablation or downregulation causes a delay in repair as evidenced by distinct experimental models (Table 3). Current evidence shows that muscle Treg cells exert influence on inflammation and fibrosis mainly by IL-10 production and inducing a macrophage shift towards an anti-inflammatory profile. In addition, Treg cells act directly on the muscle tissue inducing satellite cell activation and myoblast proliferation through the production of amphiregulin (Figure 3). Similarly to Tregs from other tissues, IL-33 has been shown to be an essential cytokine for the accumulation and activation of muscle Tregs. IL-33 belongs to the IL-1 family of cytokines and is produced by fibro/adipogenic progenitors (FAPs) produced in response to injury [38,44,59]. Evidence from in vitro experiments indicates that Treg produced a delay in myotube differentiation and an increase in the number of MuSCs, thus reinforcing the role of Treg directly in the skeletal muscle, but no effect in angiogenesis, myoblast proliferation, and/or differentiation. It is important to highlight that such experiments were performed with Treg induced in vitro from lymph node T CD4^+^ lymphocytes due to difficulty in obtaining large numbers of viable muscle Treg cells [37,40]. Skeletal muscle Tregs are distinct from lymphoid-organ Tregs and Tregs from other tissues due to characteristic transcriptome and T-cell receptor (TCR) repertoire [33,38,44]. Genes encoding mainly growth factors, cytokines/chemokines, and their receptors show different expressions in muscle Tregs when compared to other Tregs [33]. However, it is still necessary to verify which local stimuli and microenvironmental factors are essential for the stimulation and expansion of muscle Treg cells. The collection of data obtained in this research clearly demonstrates an emerging need to understand the precise role of muscle Treg cells in the context of muscle injury. Strong criticism is that many of the cell therapy experiments, including in other tissues, are still carried out in vitro and/or were performed with Tregs collected from lymph nodes and spleen but not from muscle tissue. As muscle Tregs are functionally distinct to the others, techniques must be developed to specifically stimulate these cells for use in cell therapy.

There is current evidence that Treg cells participate in the repair of various tissues. Treg cells participate in the regeneration of visceral adipose tissue whose function varies between immunity and metabolism, modulating the inflammatory response and resistance to insulin upon expression of distinct markers (e.g., PPAR-γ, IL-33, BATF, IL-10, AREG) [60,61]. In lung injury caused by influenza A infection, the Treg cells participate through AREG modulation response via a TCR-independent mechanism, which may decrease damage after infection [62] and repair tissue by inducing epithelial lung proliferation after acute lung injury (ALI) [63]. Nosbaum and coworkers demonstrated that Treg cells modulation was essential to suppress the pro-inflammatory response via IFN-γ and decrease the M1 inflammatory macrophage accumulation in the skin incision injury model [64]. All findings highlight the important role of tissue-resident Treg cells in different microenvironments with different subtypes depending on the tissue and context of injury.

Understanding the mechanisms by which tissue-resident Tregs act could help the development of therapeutic strategies capable of inducing efficient tissue repair. Several approaches have been used to induce an expansion and stability of Treg cells in the damage context, such as engineering the receptor by chimeric antigen receptor (CARs) or stimulation to overexpression Foxp3 in T CD4 cells or even induction of Treg by IL-2 stimuli [65]. Some of these therapies improved the Treg response in non-obese diabetic (NOD) mice and ameliorated the autoimmunity by in vitro antigen-specific stimulation of Treg cells by dendritic cells from NOD mice [66]. Expansion in vitro of myelin basic protein (MBP)-reactive CD4^+^CD25^+^ Tregs from donor TCR transgenic mice was able to protect from experimental autoimmune encephalomyelitis [67]. Furthermore, treatment with IL-2 in low doses was able to reestablish the Treg cell niche, in addition to clinically improving patients’ clinics with vasculitis induced by the hepatitis C virus (HCV) without any significant adverse effects [68]. Despite reports on preclinical studies in autoimmune diseases with cell-based Treg therapies, the precise mechanism by which muscle Treg cells could contribute to muscle tissue repair is still poorly understood.

## 5. Conclusions

Based on the collected data from this systematic review, it is clearly evident that the challenge remains. It is necessary to better understand the precise role of muscle Treg cells and the contribution of the immune system as a whole to the process of muscle injury and aging, with the chronology and kinetics of the process for choosing a specific strategy capable of efficiently modulating the inflammatory response and improving muscle repair without causing any further damage. All the knowledge gathered will improve the expectations of the success of therapies and the survival of individuals affected by muscular dystrophies through the delay of clinical complications and morbidity.

## Figures and Tables

**Table 1:** Search strategy for studies published from the beginning of the database up to 23 April 2021.

PubMed	Number of Results
#1) (‘‘T Lymphocytes, Regulatory’’ OR ‘‘Regulatory T-Lymphocyte’’ OR ‘‘Regulatory T Lymphocyte’’ OR ‘‘T-Lymphocyte, Regulatory’’ OR ‘‘Treg Cell’’ OR ‘‘Cell, Treg’’ OR ‘‘Cells, Treg’’ OR ‘‘Regulatory T-Lymphocytes’’ OR ‘‘Regulatory T Lymphocytes’’ OR ‘‘T-Cells, Regulatory’’ OR ‘‘Treg Cells’’ OR ‘‘Regulatory T-Cells’’ OR ‘‘Regulatory T Cell’’ OR ‘‘Cell, Regulatory T’’ OR ‘‘Cells, Regulatory T’’ OR ‘‘Regulatory T Cells’’ OR ‘‘T Cell, Regulatory’’ OR ‘‘T Cells, Regulatory’’ OR ‘‘Regulatory T-Cell’’ OR ‘‘Th3 Cells’’ OR ‘‘Cell, Th3’’ OR ‘‘Cells, Th3’’ OR ‘‘Th3 Cell’’ OR ‘‘Suppressor T-Lymphocytes, Naturally-Occurring’’ OR ‘‘Naturally-Occurring Suppressor T-Lymphocyte’’ OR ‘‘Naturally-Occurring Suppressor T-Lymphocytes’’ OR ‘‘Suppressor T Lymphocytes, Naturally Occurring’’ OR ‘‘Suppressor T-Lymphocyte, Naturally-Occurring’’ OR ‘‘Suppressor T-Cells, Naturally-Occurring’’ OR ‘‘Suppressor T-Cell’’ OR ‘‘Naturally-Occurring Suppressor T-Cells’’ OR ‘‘Suppressor T Cells, Naturally Occurring’’ OR ‘‘Suppressor T-Cell, Naturally-Occurring’’ OR ‘‘T-Cell, Naturally-Occurring Suppressor’’ OR ‘‘T-Cells, Naturally-Occurring Suppressor’’ OR ‘‘Tr1 Cells’’ OR ‘‘Tr1 Cell’’ OR ‘‘Foxp3’’)	78,222
#2) (‘‘muscle injury’’ OR ‘‘muscle diseases’’ OR ‘‘Muscles, Skeletal’’ OR ‘‘Skeletal Muscles’’ OR ‘‘Muscle, Voluntary’’ OR ‘‘Muscles, Voluntary’’ OR ‘‘Voluntary Muscle’’ OR ‘‘Voluntary Muscles’’ OR ‘‘Skeletal Muscle’’ OR ‘‘Soleus Muscle’’ OR ‘‘Muscle, Soleus’’ OR ‘‘Plantaris Muscle’’ OR ‘‘Muscle, Plantaris’’ OR ‘‘Anterior Tibial Muscle’’ OR ‘‘Muscle, Anterior Tibial’’ OR ‘‘Tibial Muscle, Anterior’’ OR ‘‘Gastrocnemius Muscle’’ OR ‘‘Muscle, Gastrocnemius’’))	610,824
#3) (Repair OR Regenerations OR ‘‘Endogenous Regeneration’’ OR ‘‘Regeneration, Endogenous’’ OR Reparation	738,780
#4) (#1) AND (#2) AND (#3)	81
**Embase ^®^**	
(‘muscle injury’/exp OR ‘injury, muscle’ OR ‘muscle damage’ OR ‘muscle injury’ OR ‘muscle lesion’ OR ‘muscle trauma’ OR ‘muscular injury’ OR ‘skeletal muscle damage’ OR ‘trauma, muscle’ OR ‘muscle disease’/exp OR ‘fibromuscular disease’ OR ‘muscle defect’ OR ‘muscle disease’ OR ‘muscle disorder’ OR ‘muscle pathology’ OR ‘muscular disease’ OR ‘muscular diseases’ OR ‘muscular disorder’ OR ‘neuromuscular manifestations’) AND (‘regulatory t lymphocyte’/exp OR ‘t lymphocytes, regulatory’ OR ‘t regulatory cell’ OR ‘t regulatory cells’ OR ‘t regulatory lymphocyte’ OR ‘t-lymphocytes, regulatory’ OR ‘tr1 cell’ OR ‘tr1 cells’ OR ‘treg’ OR ‘tregs’ OR ‘immunoregulatory t cell’ OR ‘immunoregulatory t cells’ OR ‘immunoregulatory t lymphocyte’ OR ‘regulatory t cell’ OR ‘regulatory t cells’ OR ‘regulatory t lymphocyte’ OR ‘regulatory t lymphocytes’ OR ‘regulatory t-lymphocytes’ OR ‘transcription factor foxp3’/exp OR ‘foxp3 protein’ OR ‘foxp3 transcription factor’ OR ‘forkhead box p3 protein’ OR ‘forkhead box protein p3’ OR ‘protein foxp 3’ OR ‘protein foxp3’ OR ‘scurfin’ OR ‘transcription factor foxp3’ OR ‘foxp3 regulatory t lymphocyte’/exp OR ‘foxp3 t lymphocyte’/exp OR ‘foxp3 regulatory t cell’/exp OR foxp3) AND (‘muscle regeneration’/exp OR ‘muscle regeneration’ OR ‘muscular regeneration’ OR ‘regeneration, muscle’ OR ‘muscle repair’/exp)	33

**Table 2:** Treg levels and their characteristics in muscular injuries.

Author	Species	Injury Model	Treg	Treg Characteristics
Burzyn D. (2013) [33]	Mouse	Cardiotoxin	Increased	Clonal expansion, increase in proliferation, and muscle Treg after 4 days post-injury. Muscle Treg cells released Amphiregulin growth factor, which acts directly on muscle MuSC in vitro and improved muscle repair in vivo
		Cryo-injury	Increased	Clonal expansion, increase in proliferation, and muscular Treg after 8 days post-injury
		mdx	Increased	Frequency of muscle Treg cells was increased at 4 and 12 week-old-mice compared to control mice. It was also observed clonal expansion of Treg
Villalta S.A. (2014) [39]	Human	DMD/BMD	Increased	Elevated numbers of Foxp3^+^ cells coincided with increased IL-10 expression
	Mouse	mdx	Increased	Treg was increased mainly in lesion areas in 4- and 12-week-old mice. Treg with activated phenotype and IL-10 production
Castiglioni A. (2015) [40]	Mouse	Cardiotoxin	Increased	Treg was increased since the first day post-lesion. CD3^+^CD4^+^cells have high expression of CD69 and CD25
Gazzerro E. (2015) [41]	-	-	-	Not applicable
Kuswanto W. (2016) [38]	Mouse	Cardiotoxin	Increased	Treg increases 6 days after muscle injury in young mice (2 months) but not in aged mice (6 months)
Kwee B. J. (2018) [37]	-	-	-	Not applicable
Panduro M. (2018) [44]	Mouse	Cardiotoxin	No determined	Treg cells co-localized with macrophages in the regenerating areas of injured muscle
Zhang C. (2018) [43]	Mouse	Cryo-injury	Increased	Treg cells were increased in the muscle on day 4 after lesion. Muscle Treg expresses amphiregulin, IL-10, tumor growth factor (TGF)-β, and chemokine receptors (CCR)1 and CCR5. Tregs were attracted to injured muscle by Chemokine (C-C motif) ligand (CCL)3
Cho J. (2019) [36]	Mouse	Cardiotoxin	Increased	Enhanced accumulation of muscle Tregs in both muscle-Treg TCR-Tg mice or in muscle Treg adoptive-transfer systems in RAG- mouse. The definitive muscle-Treg transcriptome was established only after Tregs migrated into the muscle
Shou J. (2021) [42]	Mouse	Contusion	Increased	Treg accumulation 3–7 days after injury

Note: DMD—Duchenne muscular dystrophy; BMD—Becker muscular dystrophy; TCR-Tg—T cell receptor-transgenic; Treg—regulatory T cell; MuSC—muscle stem cells.

**Table 3:** In vivo interventions to modulate Treg cells.

Author	Specie	Injury Model	Intervention	Treg	Outcomes
Burzyn D. 2013 [33]	Mouse	Cardiotoxin	TD injection in DTR-Foxp3 model	Decreased	Decreased frequency of CD45^+^ cells and increased inflammatory infiltrate and fibrosis
		Cryo-injury	TD injection in DTR-Foxp3 model	Decreased	Decreased centrally nucleated fibers
		mdx	anti-CD25 antibody	Decreased	Increased CK
			anti-IL2/IL2 complex	Increased	Decreased CK
Villalta S. A. (2014) [39]	Mouse	mdx	anti-CD25 antibody	Decreased	Increased muscular lesion, inflammatory infiltration, and IFN-γ expression
		mdx DEREG	TD injection in DTR-Foxp3 model	Decreased	Increase in IFN-γ expression and M1 macrophages
		mdx	anti-IL2/IL2 complex	Increased	Increase in IL-10 production. Decrease in inflammatory infiltrate and myofiber lesion (evidenced by detection of albumin and CK)
Castiglioni A. (2015) [40]	-	-	-		No treatment experiments
Gazzerro E. (2015) [41]	Mouse	mdx 4Cv	oATP	Increased	Reduced inflammatory infiltration associated with an increase in strength and reduced necrosis (CK decreased). Decrease in IL-6 and TGF-β expression. Increase in the number of regenerative cells evidenced by eMHC and myogenin expression. oATP can act directly on other cells of the inflammatory response, not only on Treg
Kuswanto W. (2016) [38]	Mouse	Cryo-injury	Mice deficient in ST2 (IL-33 receptor) on Tregs	Decreased	Increased muscle infiltrate. Decreased mean cross-sectional area of regenerating (centrally nucleated) myofibers in mice lacking ST2, specifically on Treg cells
			Restored Treg levels in the muscle of aged mice by intramuscular injection of IL-33	Increased	Histologic analysis evidenced improvement in regeneration in old mice supplemented with IL-33. There were elevated numbers of regenerating, centrally nucleated myofibers with a higher average myofibril cross-sectional area
Kwee B. J. (2018) [37]	Mouse	Ischemia in BL.CD4 KD	Intramuscular injection of alginate hydrogel with conditioned medium from conventional Treg induced in vitro	Not detected	No effect on angiogenesis and myogenesis in vivo
Panduro M. (2018) [44]	Mouse	Cardiotoxin	TD injection in DTR-Foxp3 model	Decreased	Induced an increase in IFN-γ production by NK and effector T cells. Induced macrophage dysregulation. The impact on IFN-γ production was high when Treg cells were depleted early after injury. IFN-γ inoculation mimicked Treg depletion, with increased fibrosis and inflammation
Zhang C. (2018) [43]	-	-	-		No treatment experiments
Cho J. (2019) [36]	Mouse	mdx	mdx mice backcrossed with muscle-Treg TCR-Tg mice	Increased	Leukocyte infiltration and macrophage phenotypes in muscular injury were not significantly different in Tg^+^ and Tg^−^ mdx littermates at 4 and 12 weeks. However, 12-week-old Tg^+^ mdx mice showed an increase in regenerating myofibers by histological analysis
Shou J. (2021) [42]	Mouse	Contusion	Knockout PD-1 Mice	Decreased	Reduction of the macrophage pro-inflammatory-to-anti-inflammatory switching. Downregulation of contused skeletal muscle regeneration with mitigation of muscle regeneration factors, prolonged inflammatory response period, and exacerbated oxidative stress

Note: TD—toxin diphtheria; DTR—diphtheria toxin receptor; CK—creatine kinase; DEREG—depletion of regulatory T cell; oATP—periodate-oxidized adenosine triphosphate; eMHC—myosin heavy chain-embryonic; PD-1—programmed cell death protein-1; Tg—transgenic; TCR-Tg—T cell receptor transgenic.

**Table 4:** In vitro experiments showing Treg functions related to skeletal muscle repair.

Author	In Vitro Analysis
Burzyn D. (2013) [33]	MuSCs of cardiotoxin-injured muscle from DT-treated DTR mice had decreased colony-forming capacity
Villalta S. A. (2014) [39]	No experiments
Castiglioni A. (2015) [40]	iTreg from lymph nodes induced increased MuSCs numbers and a delay in differentiation in myotubes
Gazzerro E. (2015) [41]	No experiments
Kuswanto W. (2016) [38]	No experiments
Kwee B. J. (2018) [37]	Conditioned medium from induced Treg showed no effect or no substantial effect on angiogenesis and myoblast proliferation and differentiation
Panduro M. (2018) [44]	No experiments
Zhang C. (2018) [43]	CCL3, a chemokine highly expressed in muscle after cryo-injury, attracted conventional Tregs in transwell plates. Anti-CCR1 antibody inhibited Treg recruitment significantly by CCL3
Cho J. (2019) [36]	No experiments
Shou J. (2021) [42]	No experiments

Note: TD—toxin diphtheria; DTR—diphtheria toxin receptor; iTreg—induced regulatory T cell; MuSCs—muscle stem cells.
